# A soft sensing method of billet surface temperature based on ILGSSA-LSSVM

**DOI:** 10.1038/s41598-022-26478-3

**Published:** 2022-12-19

**Authors:** Jun Liu, Luying Yang, Xinhao Nan, Yifan Liu, Qingming Hou, Kun Lan, Feng Yang

**Affiliations:** 1 College of Mechanical Engineering, Quzhou University, Quzhou, 324000 Zhejiang China; 2 School of Geophysics and Measurement-Control Technology, East China University of Technology, Nanchang, 330013 Jiangxi China; 3 College of Civil Engineering and Architecture, Quzhou University, Quzhou, 324000 Zhejiang China

**Keywords:** Engineering, Mechanical engineering, Computer science

## Abstract

It is difficult to measure the surface temperature of continuous casting billet, which results in the lack of important feedback parameters for further scientific control of the billet quality. This paper proposes a sparrow search algorithm to optimize the Least Square Support Vector Machine (LSSVM) model for surface temperature prediction of the billet, which is further improved by Logistic Chaotic Mapping and Golden Sine Algorithm (Improve Logistic Golden Sine Sparrow Search Algorithm LSSVM, short name ILGSSA-LSSVM). Using the Improved Logistic Chaos Mapping and Golden Sine Algorithm to find the optimal initial sparrow population, the value of penalty factor $\gamma$ and kernel parameter $\sigma$ for LSSVM are calculated. Global optimization method is adopted to find the optimal parameter combination, so that the negative influence of randomly initializing parameters on the prediction accuracy would be reduced. Our proposed ILGSSA-LSSVM soft sensing model is compared respectively with traditional Least Square Support Vector Machine, BP neural network and Gray Wolf optimized Least Square Support Vector Machine, results show that proposed model outperformed the others. Experiments show that the maximum error of ILGSA-LSSVM soft sensing model is 3.85733 °C, minimum error is 0.0174 °C, average error is 0.05805 °C, and generally outperformed other comparison models.

## Introduction

During the process of continuous casting production, the secondary cooling control, which is the essential part of the billet solidification process, largely determines the billet structure and defects. The use of a reasonable secondary cooling system to obtain a suitable solidification rate is the key to ensure an efficient continuous casting process and thus the good quality for billets.. The billet surface temperature, as an important feedback parameter reflecting the intensity of secondary cooling is a prerequisite for the dynamic control of secondary cooling water. Oftentimes, unreasonable pouring process and secondary cooling water distribution system could lead to quality defects inside the billet. In order to minimize the billet defects, the solidification process must be controlled. If the surface temperature of billet can be accurately obtained and feed back to the secondary cooling control system for process optimization, then the dynamic optimization of the secondary cooling water distribution and the closed-loop control of the billet temperature field can be accomplished, which is of great significance for the secondary cooling control, improving the quality of the billet, reducing the internal cracks of the billet and improving the automation level of the continuous casting process.

Due to the influence from the high temperature as well as the water mist environment of the secondary cooling zone, it is difficult to measure temperature of the moving billet^1^ using conventional contact temperature measurement methods. The presence of water film and randomly peeled iron oxide on the surface of the billet, as well as the varying surface emissivity of the billet would all make the single-point thermometer and thermal imaging camera (based on the infrared temperature measurement mechanism) unable to functionally and accurately operate during a lasting time period^2–4^ The temperature field based on mechanism model is an important research topic of billet temperature analysis. N. K. Nath, et al.^5–10^ achieved the acquisition of billet temperature by establishing the heat transfer mechanism models of billet in metallurgical process. However, continuous casting process is a complex process both physically and chemically, and it has been a difficult task to obtain model parameters during actual production process. Theoretical models are sophisticated on paper and yet cannot be guaranteed in real-life application. Shu Fuhua, et al.^11^ used the Least Squares Support Vector Machine(LSSVM) to predict the billet temperature, but the prediction still has room for improvement. Sun Jie, et al.^12^ established the billet surface temperature prediction model with the Ant Colony Algorithm, but also carries the drawback from this algorithm of easily falling into the local optimal.

Aiming the the applicability and accuracy of billet surface temperature analysis and prediction, our billet surface temperature prediction model based on ILGSSA-LSSVM is proposed in this paper. In our proposed method, first a Least Square Support Vector Machine (LSSVM) regression model is established to predict the billet surface temperature; secondly, the initial weights and thresholds of the model are optimized by using the sparrow search algorithm (SSA);finally, the initialization quality of SSA is improved by using an improved logistic chaos mapping, and the global search capability of SSA is improved by using the golden sine algorithm. This paper is organized as follows: Section "Methodology: ILGSSA-LSSVM" presents the detail methodology and structure of our proposed ILGSSA-LSSVM model; In Section "Experiments, results and analysis", we conduct experiments comparing our proposed method to others related methods, and illustrate the effectiveness and good performance of our method by quantitively analyzing the experimental results. Finally, Section "Conclusion" concludes this paper.

## Methodology: ILGSSA-LSSVM

### LSSVM regression prediction model of billet surface temperature

The support vector machine (SVM) theory proposed by C. Cortes^13^ in 1995 is a machine learning model that seeks the best compromise between the learning accuracy of training samples and the ability to identify arbitrary samples based on the VC dimension theory and the principle of minimum structural risk. SVM has strong generalization, compatible to solve nonlinear problems and can avoid local minima in solving small sample training set.

In 1999, J.A.K. Suykens^14^ proposed the least squares support vector machine (LSSVM). Based on the original method, the two norms are used and the inequality constraint is changed into equality constraint, so that solving the convex quadratic programming problem is transformed into solving linear equations, and the efficiency is improved.

The mathematical description of LSSVM is as follows: Suppose there is a training data set composed of $N$ samples,$D = \left\{ {\left( {I_{i} ,Y_{i} } \right)\left| {i = 1,2,3,...,N} \right.} \right\}$,$I_{i}$ is the input value, $Y_{i}$ is the output value. LSSVM regression model can be expressed as:

1 $$Y(I) = \omega^{T} \varphi (I_{i} ) + b$$

where, $\omega$ is the weight vector, $\varphi (I_{i} )$ is a nonlinear mapping function that maps $I_{i}$ to a higher dimensional space, and $b$ is an offset quantity. In the prediction model of this paper $N = 12$. $I_{1}$ to $I_{12}$ are molten steel temperature in Tundish, inlet temperature of crystallizer, outlet temperature of crystallizer, water flow rate of crystallizer, casting billet pulling speed, temperature of secondary cooling water, water pressure of valve port from section #0 to #2, valve opening from section #0 to #2 respectively.

In order to solve the problem of partial specific points, the error variable $e_{i}$ is introduced into each sample, and the $L_{2}$-norm of the error variable is added into the original function. The LSSVM optimization problem can be translated into:

2 $$\left\{ \begin{gathered} \min_{\omega ,b,e} J(\omega ,e) = \frac{1}{2}\omega^{T} \omega + \gamma \frac{1}{2}\sum\limits_{i = 1}^{n} {e_{i}^{2} } \hfill \\ s.t.Y_{i} = \omega^{T} \varphi (I_{i} ) + b + e_{i} ,i = 1,......,N \hfill \\ \end{gathered} \right.$$

where, $\gamma$ is the penalty factor to adjust the relationship between output $Y_{i}$ and error variable $e_{i}$.

Lagrange multiplier is introduced to solve the optimization problem:

3 $$L(\omega ,b,e,\alpha ) = J(\omega ,e) - \sum\limits_{i = 1}^{n} {\alpha_{i} [\omega^{T} \varphi (I_{i} ) + b + e_{i} - Y_{i} ]}$$

where, $\alpha_{i}$ represents the Lagrange multiplier corresponding to $I_{i}$.

According to the KKT Conditions (Karush–Kuhn–Tucker Conditions), take the derivative of each variable to solve the values of $\alpha_{i}$ and $b$:

4 $$\left\{ \begin{gathered} \frac{\partial L}{{\partial \omega }} = 0 \to \omega = \sum\limits_{i = 1}^{n} {\alpha_{i} \varphi (I_{i} )} \hfill \\ \frac{\partial L}{{\partial b}} = 0 \to \sum\limits_{i = 1}^{n} {\alpha_{i} = 0} \hfill \\ \frac{\partial L}{{\partial e_{i} }} = 0 \to \alpha_{i} = \gamma e_{i} \hfill \\ \frac{\partial L}{{\partial \alpha_{i} }} = 0 \to \omega^{T} \varphi (I_{i} ) + b + e_{i} - Y_{i} = 0 \hfill \\ \end{gathered} \right.$$

For the new sample $I$, the output of the LSSVM nonlinear regression model is:

5 $$Y(I) = \sum\limits_{i = 1}^{n} {\alpha_{i} K_{ij} + b}$$

where, $K_{ij}$ is the kernel function matrix. Radial basis function (RBF) has the advantages of strong adaptability and wide application, so RBF is chosen as the kernel function of this model. So,$K_{ij} = \exp \left\{ {\left. { - \frac{{\left\| {I_{i} - I_{j} } \right\|^{2} }}{{2\sigma^{2} }}} \right\}} \right.,$ $\sigma$ is the kernel parameter.

In LSSVM regression modeling, the prediction accuracy depends on the value of penalty factor $\gamma$ and kernel parameter $\sigma$. The penalty factor $\gamma$ is used to balance accuracy and error. The larger $\gamma$ is, the smaller the error. However, the more complex the model decision function is, the more parameters it contains, which could easily cause overfitting problem. The kernel parameter $\sigma$ represents the refinement of the partition between the value and the sample. The smaller $\sigma$ is, the more complex the curves selected in the low-dimensional space are, the more finely divided the categories are, and the overfitting is also easy to occur. Therefore, this paper adopts the sparrow search algorithm improved by Logistic chaos mapping and golden sine to carry out global optimization and select appropriate values of $\gamma$ and $\sigma$.

### Hybrid improved ILGSSA algorithm

Sparrow search algorithm is a population optimization algorithm based on swarm intelligence, specifically the foraging behavior and anti-predation behavior of sparrows^15^. In foraging behavior, the population of sparrows is divided into the finder population and follower population. After foraging, those randomly selected individuals in the population turn to be the guard population. The finder population is responsible for finding the feeding area and direction, and the follower population forages with the finder population. Each sparrow could be a finder, but the ratio of finders to followers remains constant throughout the population. When the alarm value is greater than the safe value, the sparrow population will give up the current position and fly to the safe area.

In this paper, SSA is adopted to optimize the penalty factor value and kernel parameters of LSSVM, which solves the problem of low prediction accuracy due to the limitation of parameter selection.

Assume that there is a sparrow population with a number of $n$, and position of the sparrows in $m$ dimensional solution space is expressed as:

6 $$X = \left[ {\begin{array}{*{20}c} {x_{1,1} } & {x_{1,2} } & \cdots & \cdots & {x_{1,m} } \\ {x_{2,1} } & {x_{2,2} } & \cdots & \cdots & {x_{2,m} } \\ \vdots & \vdots & \vdots & \vdots & \vdots \\ {x_{n,1} } & {x_{n,2} } & \cdots & \cdots & {x_{n,m} } \\ \end{array} } \right]$$

where, $m$ is the optimal dimension of the billet surface temperature prediction model. In this model, $m = 2$.

The fitness value of the sparrows can be expressed as:

7 $$F_{X} = \left[ {f_{1} ,f_{2},...,f_{n} } \right]^{T}$$

where, $f_{i}$ is the fitness value of each sparrow, which is the sum of the mean square error of the training set and the mean square error of the test set of the model (t the total error).

**Figure 1:**
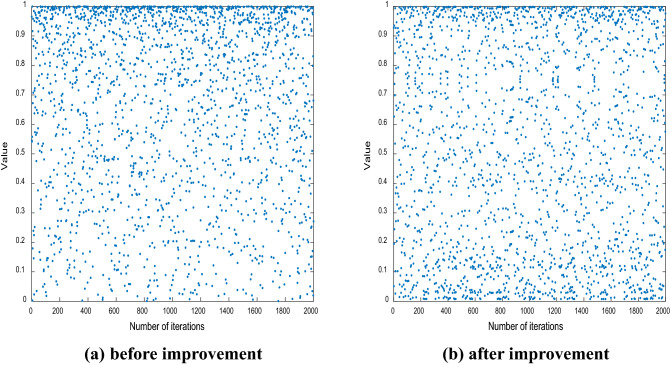
Point distribution of Logistic chaotic map before and after improvement.

Since the initial solution is generated randomly in SSA, it will cause the initial solution to be aggregated, resulting in uneven distribution of solution space. Logistic chaotic map has the advantage of decent randomness, so we use Logistic chaotic map to generate initial solution ^16–21^. However, in practical applications, the point distribution of the logistic map is more concentrated in the upper half and less common in the lower half, as shown in Fig. 1a.In order to make the mapping points more uniformly distributed and enhance the ergodicity of the chaotic map, we propose an improved Logistic chaotic map, as expressed in Eq. (8):

8 $$x_{i,j + 2} = \mu x_{i,j + 1} (1 - x_{i,j + 1} ) + (4 - \mu )x_{i,j} (1 - x_{i,j} )$$

where, $x_{i,j}\,$ $\in \left( {0,1} \right)$, is the position of the $i$ th individual in the $j$ th dimension of the initial generation of sparrow population; $x_{i,1}$ and $x_{i,2}$ are random numbers uniformly distributed on (0,1); $\mu$ is the coefficient of chaos. If $\mu$ is closer to 4, the system is more uniformly distributed on (0, 1). In this paper, $\mu$ is set to be 3.99.

**Figure 2:**
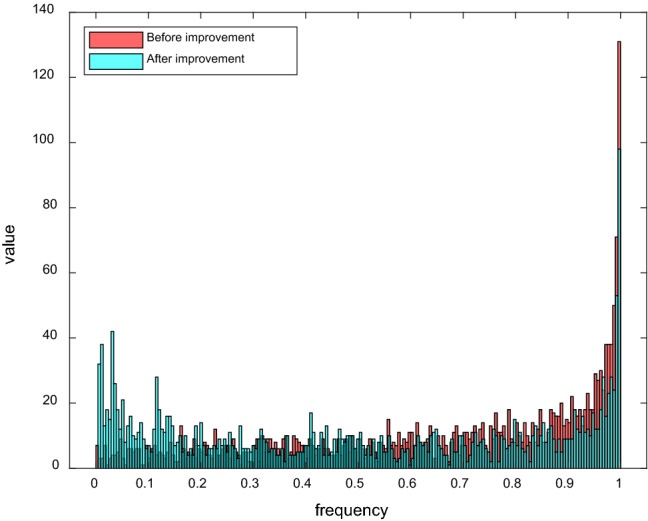
Histogram of point distribution of Logistic chaotic map before and after improvement.

In order to compare the improvement effect clearly and intuitively, we set the number of iteration times to 2000, the map points distribution after improvement is shown in Fig. 1b, and the histogram of the points distributions before and after the improvement are shown in Fig. 2.

Observing Fig. 2, we can see that before the improvement, as the chaos value increases, the mapping points are more aggregated, and the number of mapping points reaches the maximum when the chaos value reaches the maximum. The improved logistic chaos mapping has higher ergodicity and its number of points is more uniform. Therefore, the improved logistic chaos mapping is used to initialize the sparrow population and thus improve the sparrow population diversity.

Golden sine algorithm simulating the searching process of unit circle by sine function^22–25^ can obtain high quality area. In this paper, the updating rule of finders' position of sparrow population is defined as:

9 $$\left\{ {\begin{array}{*{20}c} {x_{i,j}^{t + 1} = \left\{ {\begin{array}{*{20}c} {\begin{array}{*{20}c} {x_{i,j}^{t} \cdot \left| {\sin \left( {r_{1} } \right)} \right| - r_{2} \sin \left( {r_{1} } \right) \cdot \left| {c_{1} x_{best}^{t} - c_{2} x_{i,j}^{t} } \right|} & {\begin{array}{*{20}c} {\begin{array}{*{20}c} {} & {} \\ \end{array} if} & {R_{2} < ST} \\ \end{array} } \\ \end{array} } \\ {\begin{array}{*{20}c} {x_{i,j}^{t} + Q} & {\begin{array}{*{20}c} {\begin{array}{*{20}c} {\begin{array}{*{20}c} {\begin{array}{*{20}c} {\begin{array}{*{20}c} {\begin{array}{*{20}c} {\begin{array}{*{20}c} {\begin{array}{*{20}c} {\begin{array}{*{20}c} {} & {} \\ \end{array} } & {} \\ \end{array} } & {} \\ \end{array} } & {} \\ \end{array} } & {} \\ \end{array} } & {} \\ \end{array} } & {} \\ \end{array} } & {} \\ \end{array} } & {} & {otherwise} \\ \end{array} } \\ \end{array} } \\ \end{array} } \right.} \\ {c_{1} = - \pi \left( {1 - \pi } \right) + \pi \tau \begin{array}{*{20}c} {\begin{array}{*{20}c} {\begin{array}{*{20}c} {\begin{array}{*{20}c} {\begin{array}{*{20}c} {\begin{array}{*{20}c} {} & {} \\ \end{array} } & {} & {} \\ \end{array} } & {} & {} \\ \end{array} } & {} & {} \\ \end{array} } & {} & {} & {} \\ \end{array} } & {} \\ \end{array} \begin{array}{*{20}c} {} & {} \\ \end{array} } \\ {c_{2} = - \pi \tau + \pi \left( {1 - \tau } \right)\begin{array}{*{20}c} {\begin{array}{*{20}c} {\begin{array}{*{20}c} {\begin{array}{*{20}c} {\begin{array}{*{20}c} {\begin{array}{*{20}c} {} & {} \\ \end{array} } & {} & {} \\ \end{array} } & {} & {} \\ \end{array} } & {} & {} \\ \end{array} } & {} & {} & {} \\ \end{array} } & {} \\ \end{array} \begin{array}{*{20}c} {} & {} \\ \end{array} } \\ {\tau = \frac{\sqrt 5 - 1}{2}\begin{array}{*{20}c} {\begin{array}{*{20}c} {\begin{array}{*{20}c} {\begin{array}{*{20}c} {\begin{array}{*{20}c} {\begin{array}{*{20}c} {} & {} \\ \end{array} } & {} & {} \\ \end{array} } & {} & {} \\ \end{array} } & {} & {} \\ \end{array} } & {} & {} & {} \\ \end{array} } & {} \\ \end{array} \begin{array}{*{20}c} {} & {} \\ \end{array} \begin{array}{*{20}c} {\begin{array}{*{20}c} {} & {} \\ \end{array} } & {} \\ \end{array} } \\ \end{array} } \right.$$

where, $$x_{i,j}^{t}$$ is the position of the $$i$$ th individual in the $$j$$ th dimension of sparrow population in the $$t$$ th generation; $$x_{best}$$ is the global optimal position; $$Q$$ is a random number that obeys standard normal distribution; $$r_{1}$$ and $$r_{2}$$ are random numbers obeying uniform distribution on $$\left[ {0,2\pi } \right]$$ and $$\left[ {0,\pi } \right]$$ respectively; $$c_{1}$$ and $$c_{2}$$ are partition coefficients; $$\tau$$ is golden ratio; $$R_{2}$$ is the early warning value obeying the random number of uniform distribution on $$\left[ {0,1} \right]$$; $$ST$$$$\in \left[ {0.5,1} \right]$$ is the safe value.

When $R_{2} < ST$, the early warning value is less than the safety value, the finder population is in a safe state and it searches for food in a wide area around the current location; when $R_{2} \ge ST$, the early warning value exceeds the safe value, the finder population leaves the current location and moves to another place randomly obeying standard normal distribution.

The follower population position updating rule in the population is defined as:

10 $$x_{i,j}^{t + 1} = \left\{ {\begin{array}{*{20}l} {Q \cdot \left| {\sin \left( {r_{1} } \right)} \right| - r_{2} \sin \left( {r_{1} } \right) \cdot \left| {c_{1} x_{worst}^{t} - c_{2} x_{i,j}^{t} } \right|\begin{array}{*{20}c} {} & {\begin{array}{*{20}c} {if} & {i > \frac{n}{2}} \\ \end{array} } \\ \end{array} } \\ {x_{i,j}^{t + 1} + \left| {x_{i,,j}^{t} - x_{p}^{t} } \right| \cdot A^{ + } \cdot L\begin{array}{*{20}c} {\begin{array}{*{20}c} {\begin{array}{*{20}c} {\begin{array}{*{20}c} {} & {} \\ \end{array} } & {} \\ \end{array} } & {} \\ \end{array} } & {} & {otherwise} \\ \end{array} } \\ \end{array} } \right.$$

where, ${\text{ x}}_{worst}$ is the global worst position; $x_{p}^{t}$ is the current best position of the finder population; $A$ is a $1 \times m$ matrix in which each element is randomly assigned a value of 1 or − 1, $A^{ + } = A ^{T} \left( {AA^{T} } \right)^{ - 1}$

When $i > \frac{n}{2}$, it means that the current position of the finder population is not good, there is not enough food, and the follower population will fly to another region. When $i \le \frac{n}{2}$, the follower population forages near the finder population.

The initial population randomly selects 10% to 20% of individuals as guards who are responsible for early warning and detection of the surrounding environment:

11 $$x_{i,j}^{t + 1} = \left\{ {\begin{array}{*{20}l} {x_{best}^{t} + \beta \cdot \left| {x_{i,j}^{t} - x_{best}^{t} } \right|\begin{array}{*{20}c} {\begin{array}{*{20}c} {} & {if} \\ \end{array} } & {f_{i} > f_{g} } \\ \end{array} } \\ {x_{i,j}^{t} + K \cdot \frac{{\left| {x_{i,j}^{t} - x_{worst}^{t} } \right|}}{{\left( {f_{i} - f_{w} } \right) + \varepsilon }}\begin{array}{*{20}c} {} & {if} & {f_{i} = f_{g} } \\ \end{array} } \\ \end{array} } \right.$$

where, $\beta$ is a random number that obeys standard normal distribution; $K$ is a random number that obeys the uniform distribution on [− 1,1]; $f_{i}$ is the fitness of the current individual sparrow; $f_{w}$ and $f_{g}$ are the current global worst fitness and best fitness respectively. ${\upvarepsilon }$ is an infinitesimal constant.

If $f_{i} > f_{g}$, the population feels danger and approaches to the safe position, and if $f_{i} = f_{g}$, the population stays in a safe position and moving around it.

When the sparrow population reaches the minimum fitness or the maximum number of iterations, the population stops updating.

### ILGSSA-LSSVM algorithm flow

**Figure 3:**
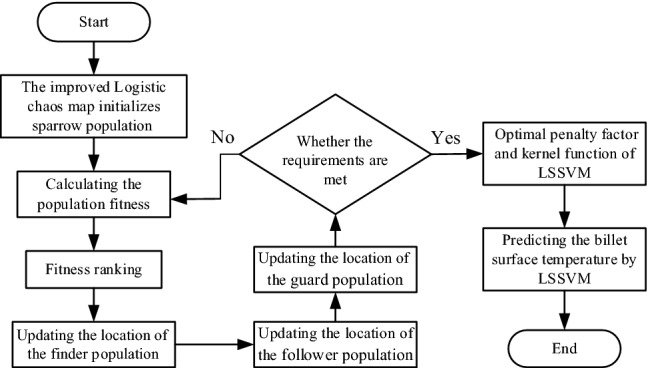
Flow chart of ILGSSA-LSSVM algorithm.

In this paper, the fitness (i.e. the total error) is used to evaluate the global optimization results. When the sparrow population reaches the minimum fitness or the maximum number of iterations, the population stops updating, and the optimal position information output is the optimal value of $\gamma$ and $\sigma$ of the LSSVM surface temperature prediction model. The flow of ILGSSA-LSSVM algorithm is shown in Fig. 3, which includes the following 6 steps:

1. The improved Logistic chaotic mapping initializes sparrow population.
2. Calculate and sort the individual fitness of the population, and mark the best fitness and the worst fitness.
3. Update the position of the finder population, follower population and guard population.
4. Determine whether the minimum fitness or the maximum number of iterations has been achieved. If not, go to Step 2). If yes, proceed forward.
5. Assign the optimal individual position of output to the $\gamma$ and $\sigma$ values of the LSSVM.
6. Predict the billet surface temperature using LSSVM regression model.

**Figure 4:**
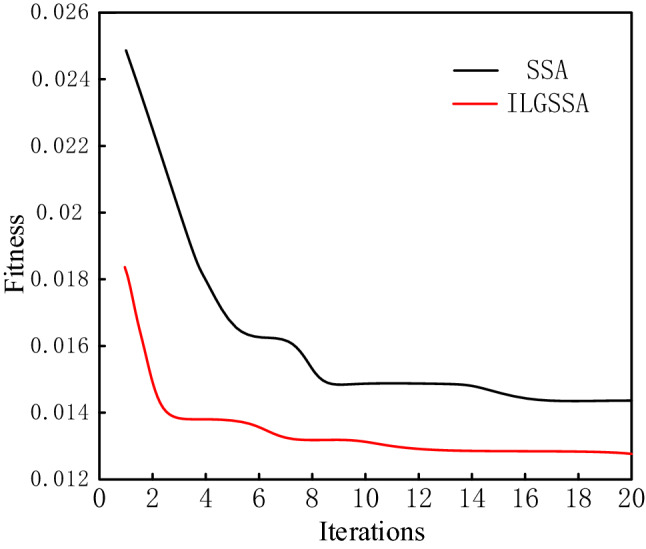
Comparison of fitness curves between ILGSSA and SSA.

A comparison of the fitness curves between ILGSSA-LSSVM model and SSA-LSSVM model over the course of 20 iterations is shown in Fig. 4. The fitness of ILGSSA tends to be stable and converges to 0.01273 at 12th generation, while the SSA tends to be stable and converges to 0.01467 at 18th generation. Compared with SSA-LSSVM model, ILGSSA-LSSVM model not only converges faster, but also has smaller fitness.

## Experiments, results and analysis

### Experiment description

**Figure 5:**
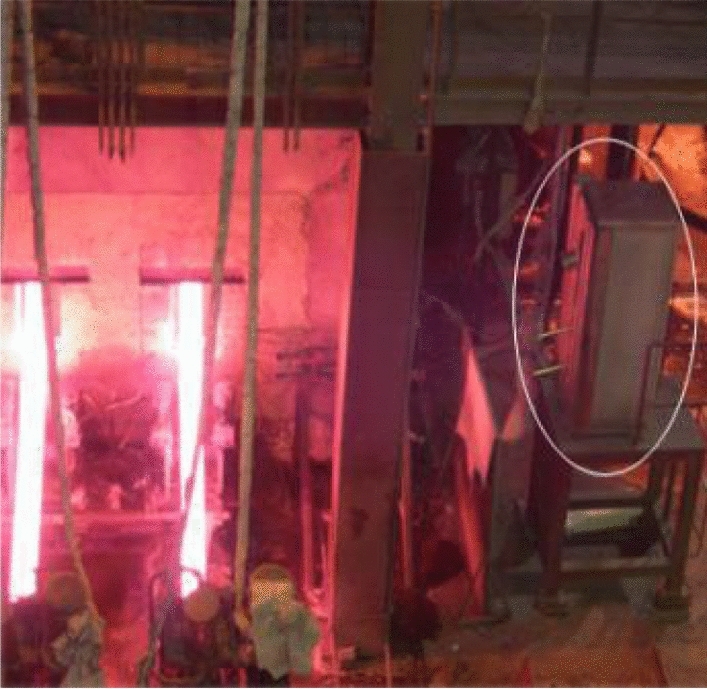
Temperature measurement verification of billet surface at secondary cooling outlet.

Figure 5 shows the infrared colorimetric thermometer used at the exit site at secondary cooling outlet. The thermometer measurements were used as the final temperature of the billet surface after emissivity correction and data filtering. In this paper, the calculated results of the soft sensing method are verified by the temperature measured by the thermometer. Proposed method is tested in the field of Fujian Sanming Steel Co., Ltd with steel grade Q235 billet in size 150 mm × 150 mm. So far, the model has been running well in the continuous casting site and meets the production requirements of the company. A total of 255,000 pieces of data during 30 days from June 1 to June 30, 2022 were actually used in this experiment and divided into 850 groups according to production time. Each group contains 300 pieces of production data, 200 of which are randomly selected as the training set and the remaining 100 as the test set.The Gray Wolf Optimizer (GWO)^26^ and SSA are both bioheuristic algorithms that emerged in recent years.. The GWO algorithm is compared with the ILGSSA algorithm. BP network is one of the most widely used neural network modelsthat can learn and store a large number of mapping relations. Here BP neural network is compared with LSSVM. In order to verify the accuracy of this soft sensing method for billet surface temperature, five soft sensing methods including ILGSSA-BP model, SSA-LSSVM model, LSSVM model, GMO-LSSVM model and BP model, will be compared respectively with ILGSSA-LSSVM model.

The parameters of ILGSSA-LSSVM model, ILGSSA-BP model and SSA-LSSVM model are set to be consistent. The parameter values of other control models are: in the SSA-LSSVM model, $\gamma = 7.4818$ and $\sigma^{2} = 6.1418$; in the GWO-LSSVM model, $\gamma = 8.1298$ and $\sigma^{2} = 3.3753$;in the LSSVM model,$\gamma = 8$ and $\sigma^{2} = 0.0958$.

Suppose the warning value of sparrow population is 0.6, the finders take 70% of the population, the followers take 30%, and the guards take 40%. The optimization range of parameters $\gamma$ and $\sigma$ to be optimized is $\gamma \in \left[ {0.01,500} \right]$,$\sigma \in \left[ {0.01,100} \right]$.The optimization result is: $\gamma = 7.9982$,$\sigma^{2} = 6.6038$.

### Results and analysis

**Figure 6:**
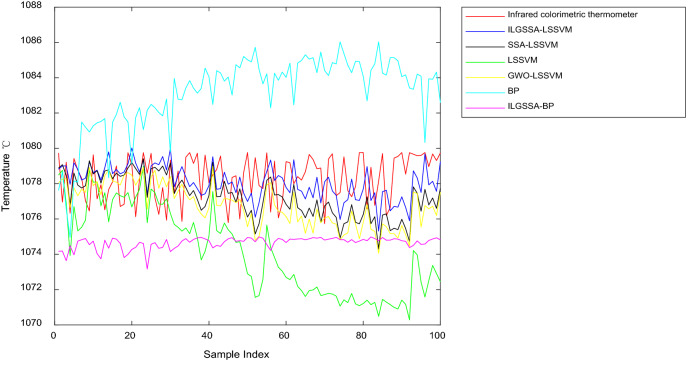
Comparison diagram of soft sensing results.

**Figure 7:**
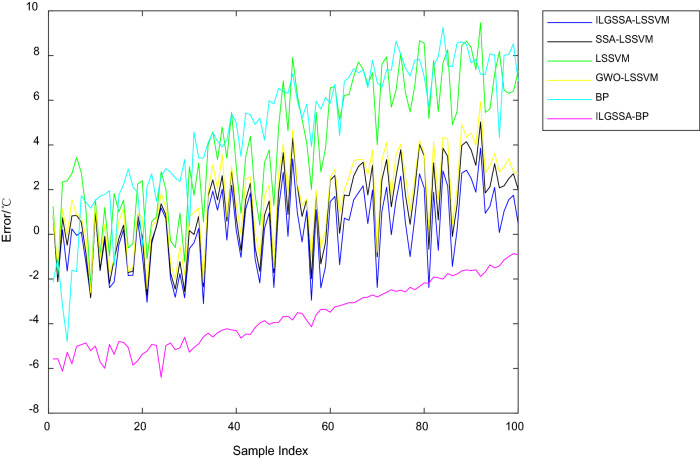
Comparison diagram of errors of various soft sensing methods.

The comparison results of the above six methods are shown in Table 1. The ILGSSA-LSSVM model has the smallest maximum error and mean error, and even the minimum error is larger than SSA-LSSVM model, but still smaller than other models. Figs 6 and 7 show the results and error comparisons for the test sets of all selected methods. Overall, the maximum error, minimum error and mean error of the LSSVM optimization model are smaller than those of the BP neural network optimization models. The ILGSSA-LSSVM model has the smallest relative error and the smallest error fluctuation, so this soft measurement method has comparatively stronger robustness than other comparison models.

**Table1:** Comparison table of soft sensing results.

Model	Maximum error/ °C	Minimum error/ °C	Mean error/ °C
ILGSSA-LSSVM	3.85733	0.01740	0.05805
ILGSSA-BP	6.39868	0.86509	3.67250
SSA-LSSVM	5.02431	0.00585	1.05740
GWO-LSSVM	5.318245	0.07711	0.64137
LSSVM	9.47212	0.28742	4.17160
BP	9.26264	0.36246	4.79316

The mean square deviation ($MSE$) and root mean square error ($RMSE$) are usually used to characterize the degree of dispersion between the predicted value and true value. The decision coefficient $R^{2}$ is used to indicate the goodness of fit of the predicted value to the true value,.The closer it is to 1, the closer the predicted value is to the true value. The mean absolute error ($MAE$) is used to evaluate the fitting accuracy to avoid biases cancelling each other out.

10 $$\left\{ \begin{gathered} MSE = \frac{1}{n}\sum\limits_{i = 1}^{n} {\left( {y_{i} - \widehat{{y_{i} }}} \right)}^{2} \hfill \\ RMSE = \sqrt {\frac{1}{n}\sum\limits_{i = 1}^{n} {\left( {y_{i} - \widehat{{y_{i} }}} \right)}^{2} } \hfill \\ R^{2} = 1 - \frac{{\sum\limits_{i = 1}^{n} {\left( {y_{i} - \widehat{{y_{i} }}} \right)}^{2} }}{{\sum\limits_{i = 1}^{n} {\left( {y_{i} - \overline{y} } \right)}^{2} }} \hfill \\ MAE = \frac{1}{n}\sum\limits_{i = 1}^{n} {\left| {y_{i} - \widehat{{y_{i} }}} \right|} \hfill \\ \end{gathered} \right.$$

where, $n$ is the number of members of the test set; $y_{i}$ is the $i$ th predicting value; and $\widehat{{y_{i} }}$ is the real predicted value of $y_{i}$.

Evaluation indexes of different models are shown in Table 2. Compared with the other five models, ILGSSA-LSSVM model has the smallest $MSE$, $RMSE$,$MAE$ and error values, and the value of $R^{2}$ is closest to 1.

**Table 2:** Comparison table of evaluation indexes of various soft sensing methods.

Model	MSE	RMSE	$R^{2}$	MAE
ILGSSA-LSSVM	1.381	1.1752	0.900	0.93262
ILGSSA-BP	15.5271	3.9404	0.41206	3.6725
SSA-LSSVM	2.0631	1.4364	0.68821	1.2684
GWO-LSSVM	1.4147	1.1894	0.6484	1.0173
LSSVM	19.884	4.4591	0.82718	4.1716
BP	29.0398	5.3889	0.56141	5.1217

In summary, the ILGSA-LSSVM soft sensing method has the highest accuracy, the best fitting degree and the smallest error fluctuation. In real life production process, the soft temperature sensing method and the infrared colorimetric thermometer verify each other, which aids and improve the calibration of temperature measurementand provides valuable reference for the actual production of steel mills.

## Conclusion

In this paper, we proposed a hybrid improved billet surface temperature soft sensing method based on ILGSSA-LSSVM, which achieved good prediction for the surface temperature of billet and provided accurate parameter feedback for secondary cooling water distribution process of continuous casting. Conclusions are as follows:

1. With the improved Logistic chaotic mapping, the stability and uniformity of the initial solution distribution of sparrow population is improved. Golden sine algorithm is used to obtain better high-quality solution region of sparrow population scanning, which improvesthe global searching ability of ILGSSA-LSSVM.
2. Global optimization method is used to find the optimal parameter combination of $\gamma$ and $\sigma$, which minimizes the negative influence from randomly initializing parameters towards the prediction accuracy.

Compared with ILGSSA-BP model, SSA-LSSVM model, LSSVM model, GMO-LSSVM model and BP model, ILGSSA- LSSVM model has the superiority of higher convergence accuracy with fewer iterations, it is able to obtain the surface temperature of billet more efficiently and accurately. We are inclined to believe that our proposed ILGSSA-LSSVM soft sensing method lays a reliable and inspiring foundation for the research of developing techniques for controlling and optimizing secondary cooling water distribution process in continuous casting of billet.

## Data Availability

The datasets used and analyzed during the current study are available from the corresponding author on reasonable request.
